# Immediate versus delayed surgery for hip fractures in the elderly patients: a protocol for a systematic review and meta-analysis

**DOI:** 10.1186/s13643-017-0559-7

**Published:** 2017-08-15

**Authors:** Thomas Klestil, Christoph Röder, Christoph Stotter, Birgit Winkler, Stefan Nehrer, Martin Lutz, Irma Klerings, Gernot Wagner, Gerald Gartlehner, Barbara Nussbaumer-Streit

**Affiliations:** 1 Department of Orthopedic Surgery and Traumatology, LK Baden-Mödling-Hainburg, Waltersdorferstraße 75, 2500 Baden, Austria; 2 Faculty of Health and Medicine, Department for Health Sciences and Biomedicine, Center for Medical Specialisations, Danube University Krems, Dr. Karl-Dorrek-Str. 30, 3500 Krems, Austria; 3 Faculty of Health and Medicine, Department for Health Sciences and Biomedicine, Center for Regenerative Medicine and Orthopedics, Danube University Krems, Dr. Karl-Dorrek-Str. 30, 3500 Krems, Austria; 4 Department of Orthopedic Surgery and Traumatology, Landeskrankenhaus Hall, Milser Straße 10, 6060 Hall in Tirol, Austria; 5 Department of Evidence-based Medicine and Clinical Epidemiology, Danube University Krems, Dr. Karl-Dorrek-Str. 30, 3500 Krems, Austria; 6 Cochrane Austria, Danube University Krems, Dr. Karl Dorrek Str. 30, 3500 Krems, Austria; 7 RTI International, 3040 Cornwallis Road, Research Triangle Park, Durham, NC 27790 USA

**Keywords:** Hip fractures, Timing of surgery, Surgical delay, Early surgery, Morbidity, Mortality, Geriatric, Elderly

## Abstract

**Background:**

Hip fractures are a major public health problem in elderly populations and are accompanied by high-mortality rates. Whether timing of surgery has an impact on morbidity and mortality has been discussed controversially, numerous studies suggest that the delay of surgery can significantly increase the risk of morbidity and mortality; others report that achieving a stable medical condition is more important than early surgery. The goal of our systematic review is to assess the impact of timing of surgery on health outcomes in patients aged 60 years or older with acute hip fracture. In addition, we will investigate differences in beneficial or harmful effects of timing of surgery in subgroups of patients based on demographic characteristics, physical status, and the use of anticoagulant medications.

**Methods:**

We will systematically search MEDLINE via Ovid, the Cochrane Library, Embase, PubMed, and clinical trial registries (from 1997 to 2017). In addition, we will search reference lists of pertinent reviews, archives of annual meetings of orthopaedic societies, and contact experts. We will include randomized controlled trials and non-randomized studies assessing the impact of timing of surgery after hip fracture in patients 60 years or older, published in English or German. Our outcomes of interest include health outcomes such as mortality, perioperative complications, functional capacity, and quality of life. We plan to perform meta-analyses if we have at least three sufficiently similar studies. If data are sufficient, we will conduct subgroup-analyses testing for differences between age groups, sex, patients’ physical status as assessed with ASA (American Society of Anesthesiologists) scores, and the use of anticoagulation.

**Discussion:**

Since this is the first systematic review on this topic since 2010, our findings will help to inform clinical practice guidelines concerning timing of surgery in hip fractures. Furthermore, our findings could contribute to define an optimal time period for surgery for different groups of patients with acute hip fracture.

**Systematic review registration:**

PROSPERO 2017 CRD42017058216

**Electronic supplementary material:**

The online version of this article (doi:10.1186/s13643-017-0559-7) contains supplementary material, which is available to authorized users.

## Background

Hip fractures are a major public health problem in elderly populations in Europe and the USA [1, 2]. In the USA, the reported incidence of hip fractures in elderly citizens is 1.1% per year [3, 4]. In Europe, the incidence ranges between 0.5 and 1.6% per year in elderly women [5–7] and about half of that risk in men [4].

Hip fractures in elderly patients are serious injuries that can lead to death, immobility, and permanent dependence, resulting in a high financial burden for health systems and societies [4, 7–9]. In senior patients, mortality rates following hip fractures range between 14 and 36% within 1 year of the injury [10–18]. Considering that life expectancy in Western countries will increase over the next decades [19–21], the burden of disease of hip fractures and their consequences will develop into an even greater public health issue in the near future.

The prognosis of elderly patients with acute hip fractures depends primarily on age, comorbidities, anticoagulation therapy, and the general physical health status [22]. In addition, mounting evidence indicates that timing of surgery might play a major role in survival after hip fracture [23–29]. Studies suggest that a delay of surgery can significantly increase the risk of morbidity and mortality in elderly patients [23–26, 30–32]. In 2010, a systematic review reported that early surgery (within 24–72 h) can reduce the risk of mortality in elderly patients by 19% [33]. These results corroborated findings of previous reviews showing that a delay of surgery beyond 48 h increased mortality within 1 year by 32% [26]. Furthermore, delayed surgery increased the risk of pneumonia [27].

Although many evidence-based guidelines recommend surgery of acute hip fracture within 48 h [34, 35], such recommendations are still controversial. Some researchers argue that delayed surgery provides valuable time for patients to achieve a better medical condition before surgery, which reduces the risk of perioperative complications, including pneumonia, deep venous thrombosis, bleeding, pulmonary embolism, urinary tract infections, and decubital ulcerations [28, 29, 36]. In clinical practice, delayed surgery of hip fractures is quite common because of a limited capacity of operating rooms or personnel, or the need for medical stabilization or anticoagulation reversal of patients before surgery [33].

The objective of our review is to systematically and objectively summarize the evidence on the impact of timing of surgery on patient-relevant health outcomes in elderly patients with acute hip fracture. In doing so, we will address several limitations of a well-conducted review by Simunovic et al. that focused on mortality [33]. In our review, we strive to address several clinically relevant questions and outcomes that were not addressed fully by the Simunovic et al. review. First, in addition to mortality, we will also focus on other patient-relevant outcomes, such as perioperative complications, functional capacity, or quality of life and aim to include studies reporting any of these outcomes. Second, if data allow, we will explore differences in benefits and harms in clinically relevant subgroups such as patients on anticoagulation treatment or patients with different American Society of Anesthesiologists (ASA) physical status classifications. Third, in addition to randomized controlled trials (RCTs), we will also include prospective controlled non-randomized studies. Non-randomized studies often provide better evidence on rare but potentially harmful events than RCTs.

## Methods

### Research questions and analytic framework

**Fig. 1:**
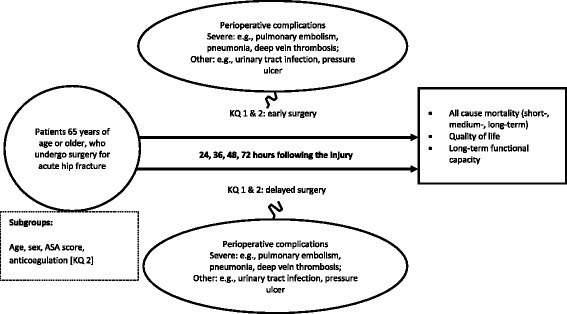
Analytic framework

Our systematic review will address the following key questions (KQ), which are also illustrated in an analytical framework (Fig. 1).

KQ 1: In patients aged 60 years or older with acute hip fracture, what is the impact of timing of the surgery on beneficial and harmful outcomes such as mortality, functional capacity, quality of life, or perioperative complications?

KQ 2: Do beneficial or harmful treatment effects of timing of the surgery vary by subgroups based on patient characteristics (age, sex), physical status (e.g., ASA Physical Status System), or common medical treatments (e.g., anticoagulation treatment)?

### Protocol and registration

We have registered our systematic review with the International Prospective Register of Systematic Reviews (PROSPERO), registration number CRD42017058216. Throughout the protocol, we followed the Preferred Reporting Items for Systematic Review and Meta-Analysis (PRISMA)-Protocols statement [37] (see Additional file 1).

### Eligibility criteria

Our population of interest are patients 60 years or older with acute hip fracture (intra- and extracapsular) who undergo surgical repair of the fracture. We will include randomized and non-randomized controlled trials (RCTs and nRCTs, respectively), and prospective controlled observational studies comparing early versus delayed surgery after acute hip fracture. Our outcomes of interest include health outcomes such as mortality, perioperative complications, functional capacity, and quality of life. Table 1 presents the inclusion and exclusion criteria.

**Table 1:** Inclusion and exclusion criteria

Category	Criteria	
	Inclusion	Exclusion
Population	• Adult (60 years or older) patients undergoing surgery for acute intra- or extracapsular hip fracture	• Adults under the age of 60 years • Patients undergoing surgery for reasons other than intra- or extracapsular hip fractures • Patients with hip fractures not related to an acute trauma • Patients with pathological fractures • Patients with periprosthetic fractures
Geography	• No limitation	• No limitation
Date of search	• Searches will go back to 1997	
Interventions	• Early surgery for hip fracture. We will use the definition for “early” as defined by authors in the primary study.	• We will exclude studies that do not compare timing of surgery
Control interventions	• Delayed surgery for hip fracture. We will include all categories of “delayed” as defined by authors in the primary study.	• We will exclude studies that do not compare timing of surgery
Outcomes	• All-cause mortality - Short term: at 1 month - Medium term: at 6 months - Long term: at 12 months • Severe perioperative complications - Pulmonary embolism - Pneumonia - Deep vein thrombosis - Others • Other perioperative complications - Urinary tract infection - Pressure ulcer - Others • Functional capacity • Quality of life (e.g., assessed with SF36)	• Studies that do not include at least one of the outcomes listed under the inclusion criteria
Publication language	• English, German	• All other languages
Study design	• Original research • Eligible study designs include: - RCTs - Prospective controlled cohort studies	• Case series • Case reports • Retrospective controlled cohort studies • Case-control studies • Studies without a control group
Publication type	Any publication reporting primary data	• Publications not reporting primary data • Publications available as abstract only

### Data source and search strategy

We will search MEDLINE (Ovid), the Cochrane Library, Embase, and PubMed (non-MEDLINE content) as well as the following trials registries: World Health Organization (WHO) International Clinical Trials Registry Platform (ICTRP) and ClinicalTrials.gov. We will limit our searches to English and German language literature, which reflects the language capabilities of our team. We will restrict the search to studies published since 1997. In addition to database searches, we will check reference lists of included articles and contact experts. An information specialist developed a comprehensive MEDLINE search strategy (see Additional file 2). The search strategy is based on a set of included studies of an existing systematic review [33] and was tested with a set of included studies of another review on this topic.

We will also handsearch the archives of annual meetings of the Orthopaedic Trauma Association, the International Society of Orthopaedic Surgery and Traumatology, the Canadian Orthopaedic Association, the European Federation of National Associations of Orthopaedics and Traumatology, the Mid-America Orthopaedic Association, the Piedmont Orthopedic Society, the Association of Bone and Joint Surgeons, the American Academy of Orthopaedic Surgeons, the Austrian Trauma Society, the Austrian Society for Orthopaedics and Orthopaedic Surgery, the German Society for Orthopaedics and Trauma, the German Society for Orthopaedics, and Orthopaedic Surgery and the German Trauma Society (all 2008–2017).

In case information about relevant outcomes or study characteristics is missing, we will contact authors of the studies and request additional information.

### Study selection

After piloting the study selection process, abstracts and full-text articles will be reviewed in two consecutive steps, by two review authors independently. Disagreements will be resolved by consensus or in consultation with a third author. We will use the software Covidence [38] to facilitate abstract/full-text selection and present the results of the study selection process using the PRISMA flowchart. We will include all records that meet our a priori defined selection criteria (see Table 1).

### Data extraction and risk of bias assessment

We will design and pilot a structured data abstraction form. Two review authors will independently extract the following information on all included studies: authors’ name, title, year of publication, study design, country, characteristics of study population (age, sex, patients’ fitness for surgery with ASA score, anticoagulant medication), sample size, type of hip fracture, timing of surgery in control and intervention group, adjustment for potential confounding, effect sizes of predefined outcomes with 95% confidence interval (CI), and counts of events for dichotomous outcomes.

Two review authors will independently assess the quality of the included studies using Cochrane Risk of Bias tool for randomized controlled trials [39] and the Newcastle Ottawa tool [40]. Disagreements will be resolved by consensus, or if necessary, in consultation with a third review author.

### Assessing the quality of the evidence

In addition, the quality of evidence will be assessed for relevant endpoints using the approach of the Grading of Recommendations Assessment, Development and Evaluation (GRADE) working group [41]. Where good-quality studies are available, the evidence will be considered to be associated with a low risk of bias. Evidence will be assessed as being consistent if the effect sizes are similar across the individual studies and pointed in the same direction. Evidence will be classed as direct when it demonstrates a direct relationship between the intervention and the health-relevant endpoint and the results of the study are applicable to the target population. It will be classed as precise when the results show a low degree of uncertainty. Finally, the quality of evidence will be classed as high, moderate, low, or very low. If the quality is high, the authors are very confident that the true effect is close to the effect estimate. In contrast, if the quality is very low, the authors assume that the true effect is likely to be significantly different from the effect estimate [42].

### Outcomes of interest

We will employ the GRADE approach to prioritize outcomes that are relevant for decision-making and for patients. Clinical experts will rate the relative importance of outcomes on a Likert scale from 1 (not relevant) to 9 (critical) via a Web-based survey using a modified Delphi approach. According to the generated mean values, we will prioritize outcomes of interest.

### Data synthesis and statistical analysis

We will consider performing meta-analyses where we have at least three unique studies of low or medium risk of bias that we deem to be sufficiently similar (in population, interventions, comparators, and outcomes). We plan to combine only studies using similar cutoffs for “early” and “delayed” surgery in a meta-analysis. Because cut-offs will vary across studies due to differences in clinical practice between centres and countries/regions, we will ask experts via a modified Delphi approach what time frames are still similar enough to be combined in a meta-analysis. We are aware of the potential biases of meta-analyses that include a small number of studies; before calculating a pooled summary estimate in a meta-analysis, we will carefully consider the heterogeneity across studies. Therefore, bodies of evidence containing fewer than three low or medium risk of bias studies or with heterogeneous or noncomparable study populations will only be used in qualitative syntheses.

For meta-analysis of trials, we will use a random-effects model. We plan to exclude studies deemed high risk of bias from our main analyses; we will include them only in sensitivity analyses. For meta-analyses of non-randomized studies, we will use generic inverse variance models to combine effects of individual studies that are adjusted for potential confounders. We will include unadjusted results only in sensitivity analyses.

To assess statistical heterogeneity in effects between studies, we will calculate the chi-squared statistic and the *I* ^2^ statistic (the proportion of variation in study estimates attributable to heterogeneity rather than due to chance [43, 44]. An *I* ^2^ from 0 to 40% might not be important; 30% to 60% may represent moderate heterogeneity; 50% to 90% may represent substantial heterogeneity; and 75% or greater represents considerable heterogeneity [39]. For the chi-squared statistic, we will adopt a *p* value of 0.1 as a threshold for clinical significance. In cases of high heterogeneity, we will explore potential reasons for heterogeneity. If we encounter high unexplained heterogeneity, we will abstain from any quantitative syntheses.

To assess publication bias, we will use funnel plots and Kendall’s tests, being aware that these tests have low sensitivity to detect publication bias, particularly with a small number of studies.

If data are sufficient, we will conduct subgroup-analyses testing for differences between age groups (60–75 years, 75.1–85 years, 85.1 years, or older), sex, patients’ physical status assessed with ASA scores, and anticoagulation.

## Discussion

Our protocol presents the methodological approach of a systematic review that will assess the effect of timing of surgery in elderly patients with acute hip fractures. In addition, the review will focus on differences of the impact of timing of surgery in subgroups such as patients on anticoagulation medication or patients with different ASA physical statuses. To our knowledge, our review will be the first study that systematically summarizes the literature on these clinically relevant questions in almost a decade.

Delays in surgeries after acute hip fracture in elderly patients are common and attributable to several factors. For example, differences between national healthcare systems and infrastructures of hospitals can influence timing of surgical interventions [30]. Specifically, in hospitals with several surgical disciplines, a competition for limited acute surgical capacities (e.g., operating rooms) may determine the timing of surgery. A comprehensive up-to-date evidence synthesis could provide the basis to prioritise early hip surgery.

Another factor contributing to delayed surgery is anticoagulant medications, particularly new oral anticoagulants (NOACs). Many patients suffering from hip fractures are treated with anticoagulant medication [45–47]. For some NOACs, no specific antidotes to block their activity are currently available [48–50]. Doctors, therefore, often prefer to wait for the effects of this medication to wear off prior to operating on the patient. Such an approach, however, is controversial because studies have claimed that patients treated with NOACs survive early surgery without any adverse health effects [51, 52]. We hope that our review will provide better insight into this clinically important matter.

Finally, various routines for assessing a patient’s health status have emerged [53, 54]. Some authors argue that patients with a poor health status must be medically stabilized before any surgery can be performed to avoid harm as a consequence of the surgical intervention [55]. To date, it remains unclear if delayed surgery is beneficial for patients with a poor physical status.

Overall, we are confident that a comprehensive summary of the best available evidence on timing of surgery for elderly patients with hip fractures will have an impact on clinical practice guidelines and will ultimately improve patient care.

## Additional files

Additional file 1: PRISMA-P 2015 checklist. (DOCX 31 kb)

Additional file 2: Search strategy. (DOCX 85 kb)

## Abbreviations

ASA: American Society of Anesthesiologists
CI: Confidence interval
e.g.: For example
GRADE: Grading of Recommendations Assessment, Development and Evaluation
ICTRP: International Clinical Trials Registry Platform
KQ: Key questions
NOACs: New oral anticoagulants
nRCT: Non-randomized controlled trial
PRISMA: Preferred Reporting Items for Systematic Review and Meta-Analysis
PROSPERO: International Prospective Register of Systematic Reviews
RCT: Randomized controlled trial
WHO: World Health Organization

## References

[1] Langley J, Samaranayaka A, Davie G, Campbell AJ (2011). Age, cohort and period effects on hip fracture incidence: analysis and predictions from New Zealand data 1974–2007. Osteoporos Int.

[2] Maalouf G, Bachour F, Hlais S, Maalouf NM, Yazbeck P, Yaghi Y (2013). Epidemiology of hip fractures in Lebanon: a nationwide survey. Orthop Traumatol Surg Res.

[3] Ettinger B, Black DM, Dawson-Hughes B, Pressman AR, Melton LJ (2010). Updated fracture incidence rates for the US version of FRAX. Osteoporos Int.

[4] Kanis JA, Oden A, McCloskey EV, Johansson H, Wahl DA, Cooper C (2012). A systematic review of hip fracture incidence and probability of fracture worldwide. Osteoporos Int.

[5] Abrahamsen B, Vestergaard P (2010). Declining incidence of hip fractures and the extent of use of anti-osteoporotic therapy in Denmark 1997–2006. Osteoporos Int.

[6] Karacic TP, Kopjar B (2009). Hip fracture incidence in Croatia in patients aged 65 years and more. Lijec Vjesn.

[7] Leal J, Gray AM, Prieto-Alhambra D, Arden NK, Cooper C, Javaid MK (2016). Impact of hip fracture on hospital care costs: a population-based study. Osteoporos Int.

[8] Marques A, Lourenco O, da Silva JA, Portuguese Working Group for the Study of the Burden of Hip Fractures in P. The burden of osteoporotic hip fractures in Portugal: costs, health related quality of life and mortality. Osteoporos Int 2015;26(11):2623-2630. 10.1007/s00198-015-3171-5 25986386

[9] Tan LT, Wong SJ, Kwek EB (2017). Inpatient cost for hip fracture patients managed with an orthogeriatric care model in Singapore. Singap Med J.

[10] Lyons AR (1997). Clinical outcomes and treatment of hip fractures. Am J Med.

[11] Panula J, Pihlajamaki H, Mattila VM, Jaatinen P, Vahlberg T, Aarnio P (2011). Mortality and cause of death in hip fracture patients aged 65 or older: a population-based study. BMC Musculoskelet Disord.

[12] Schnell S, Friedman SM, Mendelson DA, Bingham KW, Kates SL (2010). The 1-year mortality of patients treated in a hip fracture program for elders. Geriatr Orthop Surg Rehabil.

[13] Zuckerman JD (1996). Hip fracture. N Engl J Med.

[14] Morrison RS, Chassin MR, Siu AL (1998). The medical consultant’s role in caring for patients with hip fracture. Ann Intern Med.

[15] Parker M, Johansen A (2006). Hip fracture. BMJ.

[16] Haleem S, Lutchman L, Mayahi R, Grice JE, Parker MJ (2008). Mortality following hip fracture: trends and geographical variations over the last 40 years. Injury.

[17] Tolppanen AM, Taipale H, Tanskanen A, Tiihonen J, Hartikainen S (2016). Comparison of predictors of hip fracture and mortality after hip fracture in community-dwellers with and without Alzheimer’s disease––exposure-matched cohort study. BMC Geriatr.

[18] Whalen D HR, Elixhauser A. 2005 HCUP Nationwide Inpatient Sample (NIS) comparison report. HCUP method series report # 2008-01. 2008.

[19] Silvia Andueza Robustillo VC, Juchno P, Marcu M, Wronski A (2015). Eurostat demography report––short analytical Web note 3/2015.

[20] Jiaquan Xu, Sherry L. Murphy, Kenneth D. Kochanek, M.A., and Elizabeth Arias. Mortality in the United States, 2015. NCHS Data Brief, No 267. 2016. 27930283

[21] OECD (2015). Health at a glance 2015: OECD indicators.

[22] Carpintero P, Caeiro JR, Carpintero R, Morales A, Silva S, Mesa M (2014). Complications of hip fractures: a review. World J Orthop.

[23] Moja L, Piatti A, Pecoraro V, Ricci C, Virgili G, Salanti G (2012). Timing matters in hip fracture surgery: patients operated within 48 hours have better outcomes. A meta-analysis and meta-regression of over 190,000 patients. PLoS One.

[24] Leung F, Lau TW, Kwan K, Chow SP, Kung AW (2010). Does timing of surgery matter in fragility hip fractures?. Osteoporos Int.

[25] Khan SK, Kalra S, Khanna A, Thiruvengada MM, Parker MJ (2009). Timing of surgery for hip fractures: a systematic review of 52 published studies involving 291,413 patients. Injury.

[26] Shiga T, Wajima Z, Ohe Y (2008). Is operative delay associated with increased mortality of hip fracture patients? Systematic review, meta-analysis, and meta-regression. Can J Anaesth.

[27] Moran CG, Wenn RT, Sikand M, Taylor AM (2005). Early mortality after hip fracture: is delay before surgery important?. J Bone Joint Surg Am.

[28] Orosz GM, Magaziner J, Hannan EL, Morrison RS, Koval K, Gilbert M (2004). Association of timing of surgery for hip fracture and patient outcomes. JAMA.

[29] Parker MJ, Pryor GA (1992). The timing of surgery for proximal femoral fractures. J Bone Joint Surg Br.

[30] Daugaard CL, Jorgensen HL, Riis T, Lauritzen JB, Duus BR, van der Mark S (2012). Is mortality after hip fracture associated with surgical delay or admission during weekends and public holidays? A retrospective study of 38,020 patients. Acta Orthop.

[31] Smith T, Pelpola K, Ball M, Ong A, Myint PK (2014). Pre-operative indicators for mortality following hip fracture surgery: a systematic review and meta-analysis. Age Ageing.

[32] Westberg M, Snorrason F, Frihagen F (2013). Preoperative waiting time increased the risk of periprosthetic infection in patients with femoral neck fracture. Acta Orthop.

[33] Simunovic N, Devereaux PJ, Sprague S, Guyatt GH, Schemitsch E, Debeer J (2010). Effect of early surgery after hip fracture on mortality and complications: systematic review and meta-analysis. CMAJ.

[34] National GC (2014). American Academy of Orthopaedic Surgeons clinical practice guideline on management of hip fractures in the elderly.

[35] NICE (2014). Hip fracture: management, clinical guideline [CG124].

[36] Smektala R, Endres HG, Dasch B, Maier C, Trampisch HJ, Bonnaire F (2008). The effect of time-to-surgery on outcome in elderly patients with proximal femoral fractures. BMC Musculoskelet Disord.

[37] Moher D, Shamseer L, Clarke M, Ghersi D, Liberati A, Petticrew M (2015). Preferred reporting items for systematic review and meta-analysis protocols (PRISMA-P) 2015 statement. Syst Rev.

[38] Innovation VH. Covidence systematic review software: Melbourne; 2017. https://www.covidence.org.

[39] Higgins JPT GS (2011). Cochrane handbook for systematic reviews of interventions 5.1.0 [updated March 2011]: the Cochrane collaboration.

[40] Wells GABS, O'Connell D, Peterson J, Welch V, Losos M, Tugwell P. The Newcastle-Ottawa Scale (NOS) for assessing the quality of nonrandomised studies in meta-analyses. 2000. Available from: http://www.ohri.ca/programs/clinical_epidemiology/oxford.asp.

[41] Guyatt GH, Oxman AD, Vist GE, Kunz R, Falck-Ytter Y, Alonso-Coello P (2008). GRADE: an emerging consensus on rating quality of evidence and strength of recommendations. BMJ.

[42] Guyatt GH, Oxman AD, Kunz R, Vist GE, Falck-Ytter Y, Schunemann HJ (2008). What is “quality of evidence” and why is it important to clinicians?. BMJ.

[43] Higgins JP, Thompson SG, Deeks JJ, Altman DG (2003). Measuring inconsistency in meta-analyses. BMJ.

[44] Higgins JP, Thompson SG (2002). Quantifying heterogeneity in a meta-analysis. Stat Med.

[45] Doleman B, Moppett IK (2015). Is early hip fracture surgery safe for patients on clopidogrel? Systematic review, meta-analysis and meta-regression. Injury.

[46] Mattesi L, Noailles T, Rosencher N, Rouvillain JL (2016). Discontinuation of Plavix(R) (clopidogrel) for hip fracture surgery. A systematic review of the literature. Orthop Traumatol Surg Res.

[47] Soo CG, Della Torre PK, Yolland TJ, Shatwell MA (2016). Clopidogrel and hip fractures, is it safe? A systematic review and meta-analysis. BMC Musculoskelet Disord.

[48] Dager WE, Banares L (2017). Reversing the anticoagulation effects of dabigatran. Hosp Pract (1995).

[49] Riley TR, Gauthier-Lewis ML, Sanchez CK, Douglas JS (2017). Role of agents for reversing the effects of target-specific oral anticoagulants. Am J Health Syst Pharm.

[50] Arbit B, Nishimura M, Hsu JC (2016). Reversal agents for direct oral anticoagulants: a focused review. Int J Cardiol.

[51] Eikelboom J, Merli G (2016). Bleeding with direct oral anticoagulants vs warfarin: clinical experience. Am J Emerg Med.

[52] Hidalgo F, Gomez-Luque A, Ferrandis R, Llau JV, de Andres J, Gomar C (2015). Perioperative management of direct oral anticoagulant in emergency surgery and bleeding. Haemostasis monitoring and treatment. Rev Esp Anestesiol Reanim.

[53] Irlbeck T, Zwissler B, Bauer A (2017). ASA classification : transition in the course of time and depiction in the literature. Anaesthesist.

[54] Karres J, Heesakkers NA, Ultee JM, Vrouenraets BC (2015). Predicting 30-day mortality following hip fracture surgery: evaluation of six risk prediction models. Injury.

[55] Sciard D, Cattano D, Hussain M, Rosenstein A (2011). Perioperative management of proximal hip fractures in the elderly: the surgeon and the anesthesiologist. Minerva Anestesiol.

