# Supplementary material for: Immediate versus delayed surgery for hip fractures in the elderly patients: a protocol for a systematic review and meta-analysis
Source: Syst Rev. 2017 Aug 15;6:164. doi: 10.1186/s13643-017-0559-7 (PMC5558743; doi:10.1186/s13643-017-0559-7)
Supplement: Supplementary file 2 — Search strategy. (DOCX 85 kb) [file 13643_2017_559_MOESM2_ESM.docx]

**Search strategy**

Hip fractures/timing

Search draft

Ovid Medline 04 May 2017

Ovid MEDLINE(R) 1946 to April Week 4 2017, Ovid MEDLINE(R) Epub Ahead of Print May 03, 2017, Ovid MEDLINE(R) In-Process & Other Non-Indexed Citations May 03, 2017, Ovid MEDLINE(R) Daily Update May 03, 2017

| **#** | **Search** | **Results** |
| --- | --- | --- |
| 1 | exp Femoral Fractures/co, mo, su [Complications, Mortality, Surgery] | 20166 |
| 2 | ((hip or femoral or femur or pertrochanteric or subtrochanteric or intertrochanteric or intracapsular or extracapsular) adj1 fractur*).ti,ab. | 22201 |
| 3 | 1 or 2 | 33574 |
| 4 | Time Factors/ | 1113433 |
| 5 | ((delay* or time or timing or early or earlier) adj2 (surgery or surgical* or operat*)).ti,ab. | 95678 |
| 6 | ((hour* or day?) adj4 (surgery or surgical* or operat*)).ti,ab. | 75720 |
| 7 | 4 or 5 or 6 | 1254747 |
| 8 | 3 and 7 | 4388 |
| 9 | exp animals/ not exp humans/ | 4396418 |
| 10 | 8 not 9 | 4207 |
| 11 | exp age groups/ not exp aged/ | 5420379 |
| 12 | 10 not 11 | 3417 |
| 13 | (english or german).lg. | 23952988 |
| 14 | 12 and 13 | 3011 |
| 15 | limit 14 to yr="1997 -Current" | 2424 |
| 16 | remove duplicates from 15 | 2282 |
